# Testosterone/cortisol ratio: gender effect and prediction of podium results in beach sprint master rowers

**DOI:** 10.3389/fspor.2024.1466619

**Published:** 2024-12-02

**Authors:** Giovanni Ficarra, Michelangelo Rottura, Carmen Mannucci, Daniela Caccamo, Alessandra Bitto, Fabio Trimarchi, Debora Di Mauro

**Affiliations:** ^1^ Department of Biomedical Sciences, Dental Sciences, and Morpho-Functional Imaging, University Hospital, Messina, Italy; ^2^ Department of Clinical and Experimental Medicine, University Hospital, Messina, Italy

**Keywords:** cortisol, testosterone, coastal rowing beach sprint, gender, DALDA questionnaire, stress

## Abstract

**Objective:**

The purpose of this study was to address the lack of data on the stress and motivation response in master athletes during competitions, as athletic performance in this age group can be significantly impacted by stress rather than appropriate training.

**Methods:**

Coastal rowing beach sprint master athletes aged 43–57 years were examined to assess the saliva levels of stress biomarkers, cortisol, and testosterone. Specifically, samples were collected at awakening and before and after the boat race.

**Results:**

Mean values of cortisol remained unchanged from awakening and raised at the end of the competition, while testosterone levels increased before the race, suggesting an aggressive/competitive behavior. Cortisol levels were significantly higher when comparing pre-race levels with post-race (*p* = 0.001) levels and early morning with post-race (*p* = 0.006) levels. No gender effect was observed in this case. Testosterone values did not demonstrate significance when compared between time points, not even when stratifying by gender. Considering the 24 athletes, a higher testosterone/cortisol ratio was correlated with a worse podium position (B = 3.705; *p* = 0.009). When stratified by gender, the testosterone/cortisol ratio demonstrated an association with a worse outcome of the race only in female rowers (B = 4.012; *p* = 0.022). Male athletes demonstrated no significant correlation between hormone ratio and race results (B = 3.288; *p* = 0.292).

**Conclusion:**

It emerged from this study that the amateur rowers who approach competitive sport during adulthood may have problems in coping with the race-related stress and thus the outcome of their performance might be affected, as in adolescents.

## 1 Introduction

Stress, rather than proper training, can significantly impact athletic performance being responsible for metabolic and mindset variation that can impair the wellbeing of athletes. Given the considerable variability in stress hormones across all age groups and intra-individual variability, due to circadian variation, cortisol and testosterone have an important role, not only during races but also during training (1). Sport-related stress can vary among master to younger athletes. In some cases, master athletes have a long history of athletic engagement, although they may encounter unique stressors related to factors, such as aging, balancing training with other life responsibilities, and coping with the physical demands of sports over time. On the other hand, younger athletes may face stressors associated with competition, performance expectations, and establishing themselves in their athletic pursuits (2). Considering that the sources and perceptions of stress can differ across age groups and individuals, it is important to note that in the case of master athletes naïve to competitions, the above-mentioned factors relative to young athletes can be added to those specific for masters (3). In this context, factors such as training, mindset, and support systems play crucial roles in managing stress and change the perception of competition-related stress (4). Therefore, coaches have a fundamental role when amateur master athletes are taken into consideration and experience competitions for the first time (5, 6).

Coastal rowing beach sprint races are a format of coastal rowing competitions that was recently included in the Olympic Games list for Los Angeles 2028, thus gaining popularity among rowers and amateurs. These races typically involve running on the beach (approximately 10–50 m to begin and end the race) with an on-water distance of 250 m from the beach to the farthest turning buoy (buoys at approximately 85 m + 85 m + 80 m) with a 180° turn, that slows the rower speed, followed by 250 m of straight row and a last sprint once back on the beach. The combination of rowing and sand sprints (10/15 s run is approximately 6% of the race, 1:15/1:30 min row is approximately 40% of the race; 10–15 s turn, about 6% of the race; 1:15/1:30 min row is approximately 40% of the race; 10/15 s run is approximately 6% of the race) adds an exciting and physically demanding element to the competition. In this specific type of competition, quite different from the normal 2,000 m rowing race, the amateur master rowers might be more solicited than in the classical rowing setting. In addition, the typical race involves an anaerobic phase followed by a lactate tolerance phase during which the athletes need to express the maximum force (7).

Training and competition have been shown to induce distinct hormonal responses; in particular, competition is characterized by different variables that are not always present in training (8). As already demonstrated in elite athletes, cortisol levels are frequently elevated, and the testosterone/cortisol (T/C) ratio is decreased in comparison with less trained individuals during testing, training, and competition (9). Hormones can be assessed in various body fluids, including blood and saliva. The use of saliva is particularly practical for evaluations in scenarios where drawing blood under sterile conditions is not feasible, such as during races. Salivary testosterone and cortisol reflect the free fraction of the available bioactive steroid hormones found in the general bloodstream (10). High levels of free testosterone may indicate a better “bioactivity status” (11); meanwhile, low doses of testosterone are indicators of fatigue and, in some cases, of over training (12). Cortisol is recognized as the primary hormone governing catabolic processes due to its role in diminishing protein synthesis, elevating protein breakdown, and suppressing the inflammatory process, thereby influencing immune function (13). Monitoring cortisol in sports can be used as a marker of stress and stress/motivation responses to physical effort and competition (13).

Although women generally have testosterone concentrations approximately one-tenth that of men, the response of testosterone to acute physical exercise appears to increase. In untrained men, both strength and endurance exercises lead to elevated total and free testosterone levels after approximately 15–20 min of activity. The cortisol response seems to be comparable in both genders, rising in reaction to moderate exercise in both short- and long-term scenarios.

In amateur male athletes, after marathon races, transient hormonal fluctuations with a 30% decrease in the testosterone/cortisol ratio are observed. However, these hormonal changes typically return to normal in the subsequent days of intense exercise and do not align with the indicators or symptoms of overtraining (14, 15). This reaction may differ based on the athlete's fitness level. In terms of medical aspects, low testosterone levels may be indicative of poor health in men. Extreme levels of circulating androgens, whether high or low, can have negative effects on women's health. In women, the chronic hypercortisolism was found to be associated with isolated exercise-induced amenorrhea (16).

The stress relative to training and competition can also be reflected on mood. To measure this parameter, the Daily Analysis of Life Demands for Athletes (DALDA) scale (17) was developed and has been applied to assess stresses associated with the race.

In the present study, we evaluated in amateur master athletes the levels of testosterone and cortisol and the changes in their ratio. The latter was also correlated to the perceived stress and the race results of a beach sprint coastal rowing competition.

## 2 Materials and methods

### 2.1 DALDA questionnaire

This questionnaire was completed anonymously on the morning of the competition, 30 min before the official start of the races. The first part, Part A of DALDA, focuses on stress sources, encompassing factors such as diet, home life, work, friends, sport training, climate, sleep, recreation, and health. The second part, Part B, comprises items pertaining to mental and physical symptoms, such as muscle pains, boredom, irritability, general weakness, skin rashes, and congestion that may manifest as a result of the aforementioned causes of stress. Data are gathered using a 3-point Likert-type scale, where respondents can choose from three scoring options: “a” indicates “worse than usual”; “b” indicates “as usual”; and “c” indicates “better than usual.”

### 2.2 Master participants and training protocol

The study was carried out in accordance with the Helsinki declaration and approved by the rowing team coach of Rowing Club Peloro (based in Messina, Italy), the medical staff, and by the Ethics Committee of Messina University Hospital (approval protocol number 4157/2021). Written informed consent was provided from all participants to the corresponding author. A total of 24 Sicilian male and female rowers [age 50.67 ± 7.15 years; height 168.2 ± 7.03 cm; weight 65.73 ± 9.41 body mass index (BMI) 23.05 ± 2.61 kg/m^2^] participated in the study (Table 1).

**Table 1:** Characteristics of the master athletes.

	Mean ± SD
Age (years)	50.79 ± 7.41
Height (cm)	168.2 ± 7.03
Weight (kg)	65.73 ± 9.41
BMI (kg/m^2^)	23.05 ± 2.61

Of the 24 veteran athlete rowers, 6 were male and 18 were female.

The rowers were all second-year competitors. None of the enrolled participants could be considered elite athletes; they were mostly amateurs. All individuals followed a training routine of 5 days/week, based on a mesocycle preparation program. The training sessions for the races were specifically assigned 4 weeks before competitions.

In detail, before the race, athletes did five sessions of training per week, one on the ergometer and four boat training sessions with an intensive load volume.

Six weeks before the regatta, the number of boat sessions was reduced to three, with high intensity training at 75%–80% of the best training load of the athletes.

In the week before the race, the focus of training was on physiological recovery, implying less volume at 50% of their best training load. In the last days before the competition, the training sessions were relatively short but intensive: tactical situations like starts and sprints were studied with the coach, and 2 days before the race, a simulated session was dedicated to test the race distance on the boat. It is very important to simulate not only the race distance but the race trail because of the slalom around the buoys typical of coastal rowing beach sprints. In addition to the race trail in the water, the athletes must run for a variable distance (10–50 m) on the beach. At the end of the run, the athlete had to jump onto the boat. For this reason, additional skills must be developed for this particular sport.

Athletes competed in double scull boats divided according to sex. According to regulations, the athlete running on sand at the start was not the same one running at the end of the race.

### 2.3 Collection of saliva samples

Saliva collection was performed at three different time points: early morning (T0, between 6:00 and 7:30 a.m.), 5 min before the race (T1), and 15 min after the race (T2). Saliva samples were self-collected by the athletes using Salivette devices (Sarstedt Medical Systems, Genova, Italy) according to the manufacturer's instructions. All participants were instructed for sample collection as follows: the T0 sample was taken immediately after waking before getting out of bed, drinking anything, eating, and washing their mouth. In fact, the Salivette device was left on the nightstand the night before. To ensure the instructions were followed properly a tutorial video was sent to all participants via a chat group. For the next time points, the above-mentioned indications (i.e., avoiding drinking) were followed by participants; in all cases saliva sampling was through passive drool. The T1 sample was taken 5 min before the race, between 10:00 a.m. and 1:00 p.m., while the T2 sample was set at 15 min after the race. In the event of multiple competitions during the day, samples were taken in the first race.

Moreover, the athletes did not consume caffeine and/or alcohol and did not train 24 h before the race performance. Furthermore, the patients did not consume food for at least 4 h before the start of the race.

### 2.4 Biochemical tests

**Figure 1:**
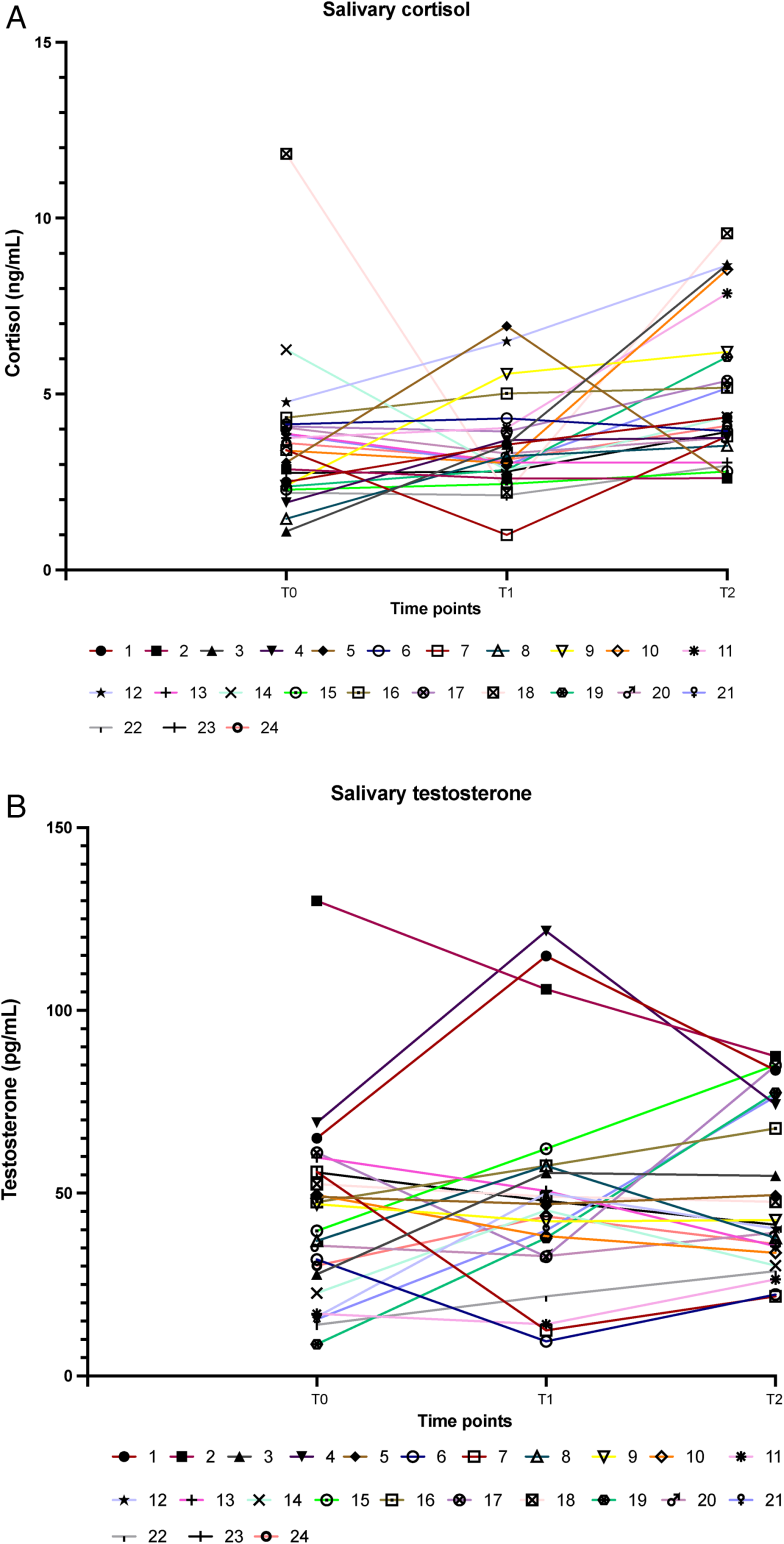
Levels of cortisol [**(A)**; ng/ml] and of testosterone [**(B)**; pg/ml] at awakening (T0), and before (T1) and after (T2) the race. Each line identifies a single athlete.

Total salivary proteins were quantified using the standard Bradford assay, and salivary cortisol and testosterone were measured using indirect competitive ELISA kits (Abcam, Prodotti Gianni, Milan, Italy) as previously reported (18). Salivary proteins were used to normalize hormone concentrations. Cortisol levels were expressed as ng/ml in all results and were transformed to nmol/L using an online tool (https://unitslab.com/node/110) to calculate the ratio with testosterone (results shown in Tables 3, 4; Figures 1A,B). Testosterone levels were always expressed as pg/ml.

### 2.5 Statistical analyses

The numerical data were expressed as means and standard deviation (SD), and the frequencies were reported as relative (%) or absolute values. The distribution of examined variables was not found normal (for cortisol and testosterone at baseline), as verified by Kolmogorov–Smirnov test. Furthermore, despite the log transformation of the study variables, the distribution remained non-normal. Then, the non-parametric Mann–Whitney *U* test was used to compare the continuous variables. The linear regression model was adopted to evaluate the association of testosterone/cortisol ratio with the podium position. Podium position was normalized for the number of entries and a further correlation with the testosterone/cortisol ratio was evaluated. Another linear regression was used to dissect out the influence of the answers of Part A on those of Part B of the DALDA questionnaire.

A *post-hoc* power calculation (*t*-test, Wilcoxon signed-rank test) was performed on cortisol saliva levels during the race, considering the median values of the 24 enrolled participants at pre- and post-race with a probability of error *α* = 0.05. The *post-hoc* power calculation was performed using G Power 3.1 (19)—including 24 enrolled participants as the sample size with median cortisol saliva levels of 3.52 ng/ml pre-race and 5.03 ng/ml (±SD = 7.68) post-race and with an α error probability = 0.05—showed an effects size d = 1.0000000 and a power (1 − β error probability) of 99.5%.

The cortisol awakening response (CAR) was calculated by subtracting cortisol levels at awakening (T0) from levels before the race (T1). A *p*-value <0.05 was considered statistically significant. All analyses were performed using the statistical package SPSS version 23.0 (IBM Corp., Armonk, NY, USA).

## 3 Results

### 3.1 Cortisol and testosterone changes in master athletes

The enrolled participants (*n* = 24) were athletes of both sexes who had been rowing for at least 2 years. They were subjected to the training protocol reported in Table 2, specifically studied to obtain the maximum performance by this specific kind of athletes who are not former sportsmen.

**Table 2:** Training protocol for outdoor race in coastal rowing.

Day	Workout
Monday	Running on the beach - 4 sets of 8 min running - 3 min rest between sets
Tuesday	Boat rowing - 4 sets of 750 m rowing on the boat - 3 min rest between sets
Wednesday	Speed training on the boat - 8 sets of 20 s rowing at race speed
Thursday	Rest day
Friday	Interval training on the rowing machine - 6–8 km total - Intervals: 1 min 30 s at 22 strokes per minute, 30 s active rest
Saturday	Boat rowing - 2 km warm-up - 4 sets of 500 m rowing at race speed - 5 min rest between sets
Sunday	Technical drills - 2 km warm-up - 1 h dedicated to technical drills only

On the day of the race, hormone levels were as depicted in Figures 1A,B and Table 3, which show individual and grouped values of either cortisol and testosterone obtained from the saliva samples of the 24 athletes.

**Table 3:** Cortisol (A) and testosterone (B) levels on the race day stratified by gender.

Timing	Cortisol (ng/ml) Total	*p*-value	Cortisol (ng/m)l M	Cortisol (ng/ml) F	*p*-value (M vs. F)
A					
Morning (T0)	3.59 ± 2.09	T0 vs. T1 0.627	3.15 ± 0.90	3.74 ± 2.37	0.790
Pre-competition (T1)	3.52 ± 1.36	T1 vs. T2 0.001	3.71 ± 1.03	3.47 ± 1.48	0.386
Post-competition (T2)	5.03 ± 2.14	T0 vs. T2 0.006	4.30 ± 1.29	5.28 ± 2.34	0.463
Timing	Testosterone (pg/ml) Total	*p*-value	Testosterone (pg/ml) M	Testosterone (pg/ml) F	*p*-value (M vs. F)
B					
Morning (T0)	43.26 ± 25.61	T0 vs. T1 0.278	62.20 ± 36.35	36.94 ± 18.17	0.110
Pre-competition (T1)	49.17 ± 28.81	T1 vs. T2 0.668	63.13 ± 39.79	44.51 ± 23.79	0.739
Post-competition (T2)	51.47 ± 22.21	T0 vs. T2 0.153	60.74 ± 23.98	48.38 ± 21.39	0.205

Values are mean ± SD of the 24 athletes.

The mean cortisol values remained unchanged between T0 and T1 and were raised at the end of the competition (T2), as shown in Table 3. On the contrary, testosterone levels increased before the race, compared with early morning levels, suggesting aggressive/competitive behavior of the athletes and remained slightly elevated after the race (Table 3). Table 3 shows no difference in the hormone levels at different time points when results are stratified by gender.

Using the Spearman correlation, a trend for men in having high cortisol with low testosterone levels was observed at T0 and vice versa (r_s_ _=_ −0.371, *p* = 0.468), while this same correlation was not observed in women (r_s_ = 0.106, *p* = 0.675). A similar observation was found at T1 (M   r_s_ = −0.429, *p* = 0.397; F  r_s_ = 0.134, *p* = 0.595) and T2 (M   r_s_ = −0.371, *p* = 0.468; F   r_s_ = 0.117, *p* = 0.645).

### 3.2 Cortisol awakening response in master athletes

Considering CAR as the difference between cortisol levels at T0 and T1, master athletes demonstrated a good ability to cope with stressors as only 2 of 24 athletes (one man and one woman) demonstrated higher levels of anticipatory stress, with a difference of more than 2.5 ng/ml in their cortisol levels compared to baseline.

**Figure 2:**
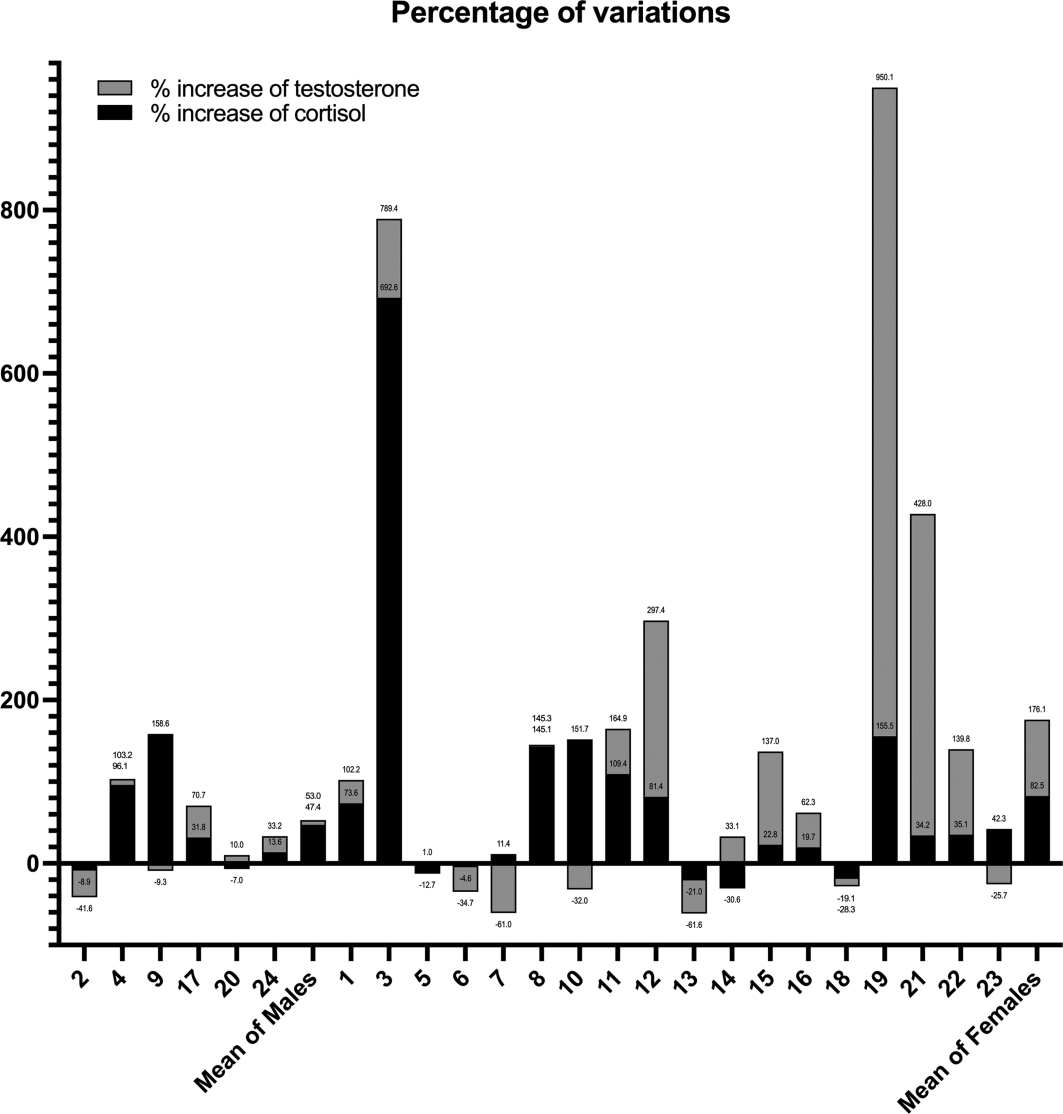
Percentage change of the cortisol and testosterone values stratified for each athlete. Each number on the *x*-axis corresponds to a specific athlete.

The graph shows that 8 of 24 athletes showed an increase in testosterone levels, and 12 of 24 showed an increase in cortisol (Figure 2). As demonstrated by the data, rowers 4, 8, 9, and 10 had an increase in cortisol levels only, while only rower 20 showed augmented levels of testosterone only (Figure 2).

### 3.3 Testosterone/cortisol ratio as podium predictor in master athletes

To better understand the influence of the hormonal response on the performance of master athletes, we interviewed the coach (Mr. Dario Femminò) to identify those who had an expected or an unexpected race result. The unexpected result could be better or worse than the actual result, but was considered unexpected by the coach given the “quality” of the competitors, regardless of gender. According to his evaluation we identified 6 of 24 participants who performed better than expected at the race (Table 4). However, no difference was observed in the values reported for the testosterone/cortisol ratio at any time point between the two groups of participants. No significance was observed between the two groups at any time point, but the small number of cases were not enough to draw conclusions.

**Table 4:** Testosterone/cortisol ratio according to expected or unexpected results.

Time point	Expected (*n* = 18)	Unexpected (*n* = 6)	*p*-value
T0	4.91 ± 3.94	6.95 ± 3.87	0.162
T1	5.48 ± 3.56	8.63 ± 6.89	0.230
T2	4.2 ± 3.14	4.97 ± 1.59	0.142

The final outcome of the race was used to divide the athletes in the two groups according to their ability to perform better or as expected.

**Figure 3:**
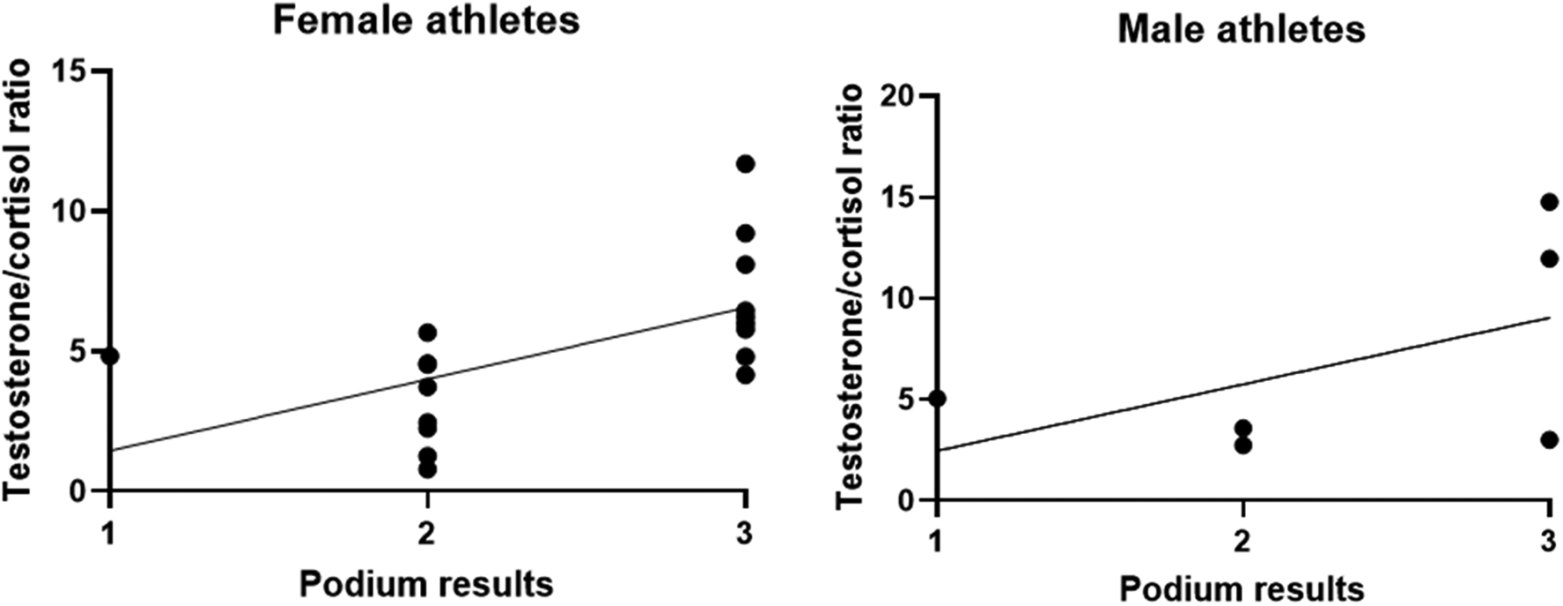
Testosterone/cortisol ratio compared to the podium results. All results are expressed as a single result per athlete.

Since the testosterone/cortisol ratio can be used as a predictor of performance, as seen in the young athletes (20), we correlated podium results with the testosterone/cortisol ratio. A higher testosterone/cortisol ratio was correlated with a worse podium position (B = 3.705; *p* = 0.009) even when stratified by gender (Figures 3A,B). When stratified by gender, the data demonstrated that in female rowers the T/C ratio was more associated with a worse outcome of the race (B = 4.012; *p* = 0.022). Male athletes demonstrated no significant correlation between hormone ratio and race results (B = 3.288; *p* = 0.292); however, it is worth noting that only 6 of 24 were men.

**Figure 4:**
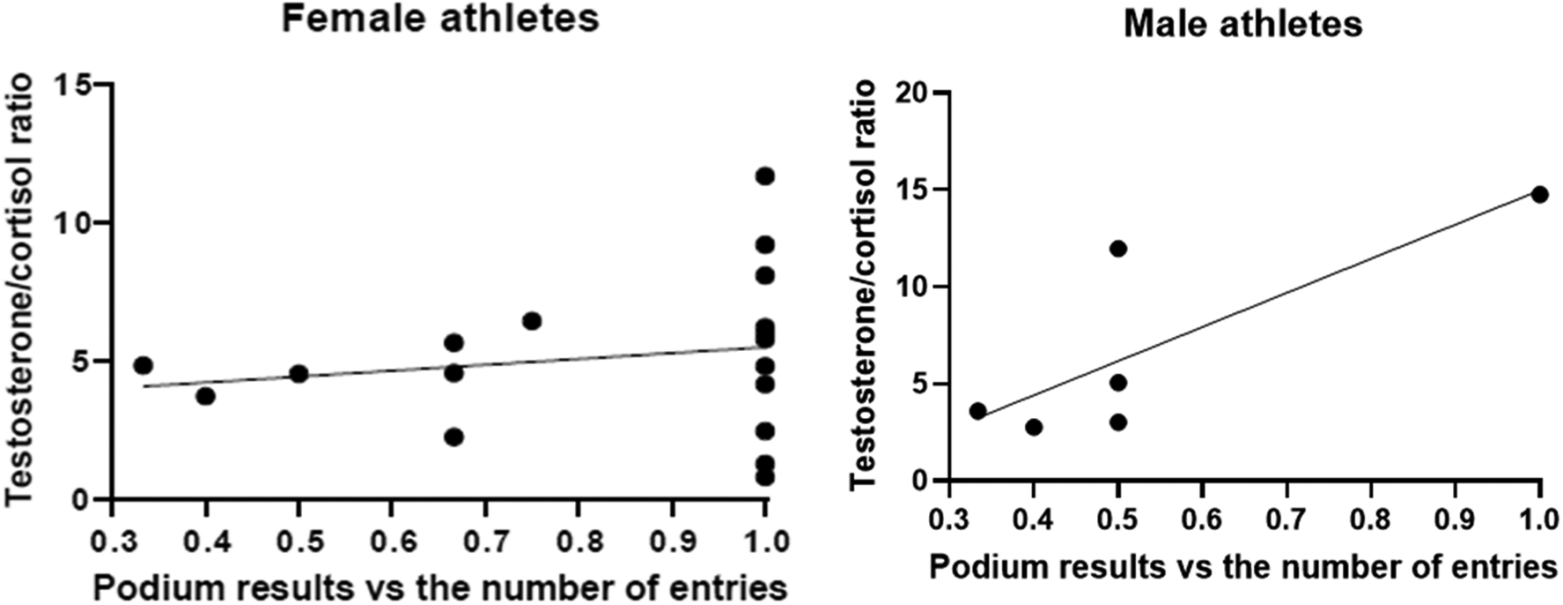
Testosterone/cortisol ratio compared to the podium results vs. the number of entries.

To further dissect these data, and considering that during master regattas there are usually few entries (even two to three teams participating in each race), we further correlated the hormonal levels with the podium result adjusted for the number of entries of each race (B = 5.414, *p* = 0.143) (Figure 4), thus no correlation was found; however, when dissecting the data by gender, a significant trend was found in male participants (B = 17.600, *p* = 0.054), while no correlation was observed in female participants (B = 5.126, *p* = 0.294).

### 3.4 Stress levels assessment

On the day of the race, the athletes completed a slightly modified version of the DALDA Questionnaire (reported in Table 5) to assess the level of perceived stress.

**Table 5:** Modified version of the DALDA questionnaire administered to master athletes.

Life demands questions (sources of stress)	A (worse than usual)	B (as usual)	C (better than usual)
How do you consider your diet?	1 (4.2%)	14 (58.3%)	9 (37.5%)
How is your home life?	1 (4.2%)	21 (87.5%)	2 (8.3%)
How do you feel about: school/university/work?	8 (33.3%)	15 (62.5%)	1 (4.2%)
How do you feel about your friends?	0	12 (50.0%)	12 (50%)
How do you feel during training?	0	7 (29.2%)	17 (70.8%)
How do you feel with this climate?	7 (29.2%)	10 (41.7%)	7 (29.2%)
How is the quality of your sleep?	9 (37.5%)	14 (58.3%)	1 (4.2%)
How is the quality of your free time?	2 (8.3%)	14 (58.3%)	8 (33.3%)
How is your health?	1 (4.2%)	18 (75.0%)	5 (20.8%)
Mood state questions (symptoms of stress)			
Do you feel muscle pains?	5 (20.8%)	15 (62.5%)	4 (16.7%)
How do you evaluate my rowing technical skills?	6 (25.0%)	16 (66.7%)	2 (8.3%)
What is your level of tiredness?	2 (8.3%)	19 (79.2%)	3 (12.5%)
How is your need for rest?	6 (25.0%)	15 (62.5%)	3 (12.5%)
How do you feel about extra workouts?	3 (12.5%)	14 (58.3%)	7 (29.2%)
How is your level of boredom?	5 (20.8%)	11 (45.8%)	8 (33.3%)
How do you rate your recovery time?	1 (4.2%)	15 (62.5%)	8 (33.3%)
How do you rate your irritability?	9 (37.5%)	14 (58.3%)	1 (4.2%)
How is your weight?	9 (37.5%)	13 (54.2%)	2 (8.3%)
Do you feel a lump in the throat thinking about the race?	11 (45.8%)	9 (37.5%)	4 (16.7%)
How do you assess your mood?	6 (25.0%)	14 (58.3%)	4 (16.7%)
Do you experience unexplained aches?	4 (16.7%)	9 (37.5%)	11 (45.8%)
How do you assess my technique strength in rowing?	1 (4.2%)	12 (50.0%)	11 (45.8%)
Do you consider your sleep adequate?	9 (37.5%)	14 (58.3%)	1 (4.2%)
How do you assess the recovery time between races?	5 (20.8%)	19 (79.2%)	0
How do you assess your general weakness?	5 (20.8%)	13 (54.2%)	6 (25.0%)
How do you assess your interest in the race?	1 (4.2%)	5 (20.8%)	18 (75.0%)
How do you assess the possibility of starting an argument in the race field?	10 (41.7%)	9 (37.5%)	5 (20.8%)
Do you usually notice skin rashes or irritation after the race?	9 (37.5%)	7 (29.2%)	8 (33.3%)
How do you assess your pre-race motivation?	1 (4.2%)	9 (37.5%)	14 (58.3%)
How do you assess your training effort before this race?	0	7 (29.2%)	17 (70.8%)
How do you assess your willingness to collaborate on the race field?	0	4 (16.7%)	20 (83.3%)
How do you assess your hydration status post-race?	6 (25.0%)	14 (58.3%)	4 (16.7%)
How do you assess your level of sociability on the race field?	1 (4.2%)	8 (33.3%)	15 (62.5%)
How do you assess the possibility of having fun at this race?	1 (4.2%)	5 (20.8%)	18 (75.0%)

A higher prevalence of “normal” response was observed for all “wellbeing questions,” with the exception of the question “how do I feel during training” in which 70.8% of athletes answered “better than normal” (Table 5).

A higher prevalence of “better than normal” was reported for the following “mood state questions”: “Do you experience unexplained aches?” (45.8%); “How do you assess your pre-race motivation?” (58.3%); “How do you assess your interest in the race?” (75.0%); “How do you assess your training effort before this race?” (70.8%); “How do you assess your willingness to collaborate on the race field?” (83.3%); “How do you assess your level of sociability on the race field?” (62.5%); and “How do you assess the possibility of having fun at this race?” (75.0%).

Moreover, a greater frequency of “worse than normal” was observed for the following questions: “Do you feel a lump in the throat thinking about the race?” (45.8%); “How do you assess the possibility of starting an argument in the race field?” (41.7%); and “Do you usually notice skin rashes or irritation after the race?” (37.5%) questions. All other “mood state questions” present a higher frequency than the “normal” response (Table 5).

In the effort to understand if the mood state had an influence on the perception of tiredness before the competition, some correlation was carried out between questions. For instance, the answer “better than normal” to the question “Do you feel muscle pains?” was significantly correlated with the response “better than normal” to the following questions: “How do you consider your diet?” (B = 0.386; *p* = 0.029); “How do you feel about school/university/work?” (B = 0.538; *p* = 0.004); and “How is the quality of your sleep?” (B = 0.489; *p* = 0.007).

The “better than normal” answer to the question “What is your level of tiredness?” was significantly correlated with the response “better than normal” to the following questions: “How is the quality of your free time?” (B = 0.278; *p* = 0.004); and “How is your health?” (B = 0.724; *p* < 0.001).

The “better than normal” answer to the question “Do you feel a lump in the throat thinking about the race?” was significantly correlated with the response “better than normal” to the following questions: “How do you feel about: School/University/work?” (B = 0.459; *p* = 0.043); “How do you feel during training?” (B = 1.194; *p* < 0.001); “How do you feel with this climate?” (B = 0.389; *p* = 0.013); and “How is the quality of your sleep?” (B = 0.787; *p* = 0.001). Conversely, the answer “better than normal” to the question “Do you feel a lump in the throat thinking about the race?” was related to the response “worse than normal” only to the question “How is the quality of your free time” (B = −0.552; *p* = 0.006).

The “better than normal” response to the question “How do you assess the possibility of starting an argument in the race field?” was directly correlated with the “better than normal” response to the question “How is the quality of your sleep” (B = 0.541; *p* = 0.028) and inversely correlated to the question “How do you feel with this climate?” (B = −0.456; *p* = 0.012).

The answer to the question “How do you assess your level of sociability on the race field?” was not significantly influenced by any of the answers given in the Life demands section.

## 4 Discussion

Beach sprint is a discipline that requires anaerobic training (40%/20%), which would include power training, lactate tolerance, and repeat sprint training. The beach sprint race is made up of 12% running, boat entry, and exit, 83% rowing, and 5% turning; all happening in less than 3 min (21). This highly demanding activity requires rapid energy adaptation and hormone-driven metabolic changes. One of the main observations deriving from this study refers to the fluctuations of the cortisol levels and the perception of stress by the amateur master rowers.

In fact, the athletes enrolled as amateurs were not used to the stress of competition from a younger age and this was clearly indicated by the increase in cortisol from waking until the end of the race (Figure 2). Cortisol should typically decrease from a morning peak to very low levels in the evening (22); for the same age group, the common values of saliva at waking are 4.5 ng/ml in women and 4.3 ng/ml in men, while in the evening these values are 0.97 and 0.76 ng/ml in women and men, respectively.

Cortisol influences metabolism by aiding in the regulation of blood glucose levels during exercise. It achieves this by influencing skeletal muscle and adipose tissue, promoting the mobilization of amino acids and lipids, and stimulating gluconeogenesis. Although cortisol levels rise during exercise, the majority of its alterations and impacts on the body occur in the initial recovery period after the exercise session (22–25). Physical exercise can be viewed as a stress model, where internal or external stressors may disrupt the organism's dynamic equilibrium or homeostasis. Responses to these stressors manifest in both physical and behavioral changes. Once a certain threshold is reached, the stress system triggers systemic responses in both the brain and peripheral elements, such as the autonomic sympathetic and the hypothalamic–pituitary–adrenal system. Almost 50 years ago, Selye (26) introduced the term “normal stress,” and in the context of sports, this typical response stimulates bodily functions and may generate hormonal responses through various pathways. This, for instance, aids in maintaining blood glucose levels during acute exercise sessions, as mentioned earlier. However, defining harmful stress in the realm of sports is not straightforward. Questions arise, such as whether a significant drop in the testosterone/cortisol ratio after marathon races, from an acute perspective, indicates normal stress or harmful stress. From a chronic standpoint, the inhibition of the hypothalamic–pituitary–gonadal axis in cases of isolated exercise-induced amenorrhea might signal harmful stress.

In the studied population, the observed increase in cortisol from awakening to the end of the race, in both male and female rowers, has to be related to the poor ability to cope with the stress of competing. Similar results were obtained in the population of adolescent athletes that was previously studied (20). Despite the clear difference in age range of the two populations, given that in both cases we have amateur athletes, it is not unexpected that the pressure and stress of competition affects hormone levels in either adolescents or adults.

The observed increases in cortisol 5 min before and 15 min after the competition do not relate to gender (Table 3A), but could either be due to the small number of male athletes enrolled in the study (only 6 of 24) or to the menopausal status (14 of 18 women were post-menopause) of the female athletes, which limits hormonal fluctuations (27). In fact, cortisol is produced not only in the adrenal glands, but also in adipose tissue, where an enzyme called 11 beta-hydroxysteroid dehydrogenase type 1 (11 beta-HSD1) converts inactive cortisone to active cortisol. Estrogen can increase the production of 11 beta-HSD1 in preadipocytes in women, so changes in estrogen levels during their fertile years vary the production of cortisol (28). However, in the studied population, body mass revealed a normal composition with a mean BMI of 23.05 ± 2.61; therefore, a contribution of 11 beta-HSD1 is likely not relevant.

As far as testosterone is concerned, the results showed great variability from morning to the end of the race but no particular differences over time or across genders (Table 3B). This was seen in many other studies that evaluated hormonal fluctuations in amateur sportsmen (29, 30). Similar to cortisol, testosterone levels exhibit a linear increase in reaction to physical exercise until a particular threshold of exercise intensity is attained, typically peaking at the conclusion of the physical activity, as observed in our athletes at the end of the race (T2). Nevertheless, certain studies indicate a negative correlation between cortisol and testosterone under specific conditions (15), although we did not observe any correlation in our rowers. Previous observations showed that athletes close to the overreaching state have no differences in cortisol levels when subjected to trials. For these reasons, monitoring cortisol levels and the stress level of an athlete in general is always of outmost importance to avoid excessive training (31).

Regular physical training can increase testosterone levels in older people, suggesting that the exercising status of master athletes can preserve testosterone levels in old age (32). The testosterone/cortisol ratio has been extensively examined in sports physiology, and during strength training, the promotion of an anabolic environment fosters protein synthesis and muscle hypertrophy, particularly in type IIb muscle fibers. Monitoring the anabolism/catabolism ratio is also crucial in endurance training to enhance the aerobic metabolism of muscles, and in both strength and endurance training, this might be related to signs and symptoms of overtraining (16).

A noteworthy correlation was discovered between the increase in testosterone resulting from physical training and the vastus lateralis cross-sectional area (33), suggesting that acute spikes in testosterone may play a crucial role in muscle hypertrophy. However, these acute responses are attenuated in women and the elderly, diminishing the hypertrophic potential in these populations (34–36). Both total and free testosterone levels have been employed to assess training volume in both men and women (37). For instance, Vervoorn et al. proposed that, unlike what is observed in professional male rowers, the free testosterone/free cortisol ratio is not a reliable indicator of the anabolic/catabolic balance in professional female rowers (38). These data suggest that a high level of “anabolic hormones” in the internal environment may not necessarily be linked to muscle or strength gains. For example, increases in testosterone after strength training could trigger a temporary physical stress response unrelated to hypertrophic gains (39). Consequently, despite some well-designed studies not correlating anabolic hormonal responses to exercise with muscle fiber gains, certain reports have indicated associations of testosterone with motivation and preparedness to compete or perform, as well as enhanced acute neuromuscular performance in athletic populations (40).

In a study involving 20 master athletes and 28 sedentary elderly men, the concentration of serum and salivary testosterone was determined, showing that changes in body composition, due to aging abrogated the positive effect of training on testosterone levels (41). This may account for the reductions in the T/C ratio, observed during aging, which can reach a 30% decrease and may also be indicative of incomplete recovery (42). From these data, it is possible to speculate that master athletes need a longer recovering period, and this new evidence from the literature points to the need of further research to develop a training model capable of reducing these testosterone and T/C ratio deficits in master athletes during the sports season. In addition, it also suggests the need for more study involving master athletes to better understand if the longer recovery period is limited to endurance sport or to all kind of sport.

In our study, the T/C ratio was not used for correlations with an overtraining status nor to muscle hypertrophy. On the contrary, it was used as a predictor of performance. Regarding the testosterone/cortisol ratio and its relationship with the expected or unexpected race results (i.e., a better or worse performance according to the evaluation of the coach Mr. Dario Femminò), we did not see any correlation, even if participants with an unexpected performance displayed higher values of the ratio (8.63 vs. 5.48), suggesting less stress or more aggressiveness.

Correlating the T/C ratio with the race outcome, at increasing values of the ratio a correlation with a worse podium positioning was observed, especially in women suggesting a gender effect that needs to be proven in larger populations.

When normalizing the podium results to the number of entries, the T/C ratio almost predicts bad performance in men compared with women. It thus seems that the stress in women is not related to the number of competitors in a race rather than the race itself.

As far as the DALDA questionnaire is concerned, it is important to point out that it is often administered at different timings to the athletes as an indirect measurement of overreaching and the efficacy of the training sessions (5, 17). In the context of continuous assessment, five of the subscales exhibited efficacy by responding promptly (non-training stress, fatigue, physical recovery, general health/wellbeing, being in shape). In addition, three other subscales (conflicts/pressure, self-regulation, lack of energy) demonstrated usefulness in monitoring long-term wellbeing (43). As previously demonstrated, the DALDA B subscale is applicable for identifying anticipated shifts in psychological stress levels following overreaching protocols, as can be perceived those used to prepare athletes for races (44, 45).

The correlations observed within the answers of Parts A and B of the DALDA questionnaire suggest that athletes who experience stress and pressure in their daily activities have a lower ability to cope with the stress arising from the competition. Unfortunately, to maintain anonymity, it was not possible to analyze questions by gender nor to correlate with the T/C ratio of each athlete, although given the observed correlation it is possible to speculate that the large majority of the enrolled participants experienced similar feelings.

The present study has some limitations. First, the relatively small sample size restricts the generalizability of the obtained data. In addition, the number of male rowers is one-third of the number of female rowers; however, despite these discrepancies, some interesting results have been obtained. Second, the lack of hormone data from training sessions might influence the validity of the results. In fact, it is known that cortisol may have an erratic distribution, especially in those athletes close to overreaching (31), as may happen when preparing for a competition. Third, participation was voluntary; therefore, expanding the sample size was not feasible. Furthermore, the gender distribution is also uneven, with only six male participants, which could influence our results. Additional limitations concerned both the unawareness of the athletes’ hydration status and the timing of pre-competition hormone sampling. In fact, athletes performed their respective competitions in a time window between 10 a.m. and 1 p.m. Therefore, such a time margin could affect the concentration of the salivary sample. It is also important to note that we did not assess the use of supplements in our master athletes, which could have an impact on performance and are also widely used during training and competitions (46).

## 5 Conclusion

It emerged from this study that the amateur rowers who approach competitive sport during adulthood may have problems in coping with the race-related stress; thus, the outcome of their performance might be affected, as in adolescents (20). In fact, the results of our study clearly indicate that elevated cortisol levels, especially in women, are responsible for a worse performance. Thus, in addition to the monitoring of hormone levels that may be expensive, the DALDA can be considered as a tool to enable coaches to strategically assess athletes’ stress status (45). The fear of failure or underperformance, along with evaluations from parents, friends, coaches, and others, can negatively impact an athlete's performance, as is often common in female athletes (47). To avoid these problems, coaches should create tailored training programs to help athletes lower their anxiety levels and improve their performance and capabilities by developing effective and efficient sports skills.

Coaches can use different methods to engage in meaningful communication with athletes, gaining insights into the athletes’ true feelings across different training periods. This can be useful for adjusting training loads on a given day, as it could be the day of a race.

As demonstrated in adolescents (48), the importance of reinforcement is integral to the teaching process, since athletes of all ages need to learn how to cope with sport-related stressors. However, the athletes’ mental balance and concentration can be disrupted by the coaches’ behavior, i.e., being anxious for race results, or providing unnecessary and excessive feedback to an athlete may result in poor performance for both the individual and the team (49).

For these reasons, a successful coach must also be a proficient listener, even in the case of adult amateurs who participate in competitions for the first time.

## Data Availability

The raw data supporting the conclusions of this article will be made available by the authors, without undue reservation.
